# Therapeutic Use of Pilocarpin in Skin Diseases

**Published:** 1880-07

**Authors:** 


THERAPEUTIC USE OF PILOCARPIN IN SKIN DISEASES.

PROFESSOR PICK, editor of the *Vierteljahrsschrift für Dermatologie und Syphilis*, gives, in the first number of his journal for the current year, some account of his experience in the use of pilocarpin in certain diseases of the skin. The drug was given in the dose of 0.01 gramme ($\frac{1}{7}$ grain) in aqueous solution, morning and evening, an hour after meals, the patient being in bed or, in summer-time, walking about. Increased salivary secretion was first observed, and a few minutes later increased perspiration. In a few cases one or the other was absent. After three or four weeks the medicine seemed to lose its effect, so that a larger dose had to be given. No evil effect was noted, and in some cases the patient's general health seemed to be improved by the medicine. In psoriasis no effect whatever seemed to be induced upon the course of the disease. In acute eczema, pilocarpin aggravated the disease, while in chronic eczema some advantage seemed to be gained by its use. In pruritus cutaneus, and particularly in one very severe case of pruritus vulvæ, pilocarpin acted favorably. In a single case of chronic and rebellious urticaria, pilocarpin, given in one-tenth-grain dose twice daily, resulted in a cure. In the case of a man suffering from well-marked alopecia areata of six months' standing, a two weeks' course of pilocarpin was followed by the appearance of fine, colorless lanugo, and by the end of twelve weeks the hair was restored. Other cases appeared to be equally favorably affected. In ten cases of alopecia "pilyroides" a favorable result was obtained: so that this remedy may be looked upon as a satisfactory one in cases where a strong hereditary tendency to baldness does not exist.

